# A Role for Androgens in Epithelial Proliferation and Formation of Glands in the Mouse Uterus

**DOI:** 10.1210/en.2015-2032

**Published:** 2016-03-10

**Authors:** Ioannis Simitsidellis, Douglas A. Gibson, Fiona L. Cousins, Arantza Esnal-Zufiaurre, Philippa T. K. Saunders

**Affiliations:** Medical Research Council Centre for Inflammation Research, The Queen's Medical Research Institute, EH16 4TJ, Edinburgh, United Kingdom

## Abstract

The endometrium consists of stromal and epithelial compartments (luminal and glandular) with distinct functions in the regulation of uterine homeostasis. Ovarian sex steroids, namely 17β-estradiol and progesterone, play essential roles in modulating uterine cell proliferation, stromal-epithelial cross-talk and differentiation in preparation for pregnancy. The effect of androgens on uterine function remains poorly understood. The current study investigated the effect of the non-aromatizable androgen dihydrotestosterone (DHT) on mouse endometrial function. Ovx female mice were given a single sc injection (short treatment) or 7 daily injections (long treatment) of vehicle alone (5% ethanol, 0.4% methylcellulose) or vehicle with the addition of 0.2 mg DHT (n=8/group) and a single injection of bromodeoxyuridine 2 hours prior to tissue recovery. Treatment with DHT increased uterine weight, the area of the endometrial compartment and immunoexpression of the androgen receptor in the luminal and glandular epithelium. Treatment-dependent proliferation of epithelial cells was identified by immunostaining for MKi67 and bromodeoxyuridine. Real-time PCR identified significant DHT-dependent changes in the concentrations of mRNAs encoded by genes implicated in the regulation of the cell cycle (*Wee1, Ccnd1, Rb1*) and stromal-epithelial interactions (*Wnt4, Wnt5a, Wnt7a, Cdh1, Vcl, Igf1, Prl8, Prlr*) as well as a striking effect on the number of endometrial glands. This study has revealed a novel role for androgens in regulating uterine function with an effect on the glandular compartment of the endometrium. This previously unrecognized role for androgens has implications for our understanding of the role of androgens in regulation of endometrial function and fertility in women.

The endometrium is a hormone-dependent multicellular tissue that consists of stromal and epithelial compartments. The stromal compartment contains mesenchymal fibroblasts, a well-developed vasculature and immune cells, whereas the epithelium is divided into a luminal and a glandular component, each with a distinct identity and role in reproductive physiology (1). Both compartments respond to hormonal cues in a coordinated spatial and temporal manner characterized by extensive cross-talk to regulate the development and homeostasis of the tissue (2–4).

Although the effects of estrogens on endometrial function have been extensively studied, androgens have only recently emerged as key players in the regulation of endometrial function. Analysis of circulating steroid hormone levels using sensitive liquid chromatography–tandem mass spectrometry has revealed significant concentrations of androgens present throughout the menstrual cycle in women, exceeding those of circulating estrogens (5). Investigation of circulating androgen levels in cycling female mice reported detectable concentrations of the circulating T metabolite 5α-dihydrotestosterone (DHT; 2.8 ± 0.4 pg/mL) during all phases of the estrous cycle, with concentrations peaking during proestrus and metestrus (6). In aging female mice, there is a significant increase of circulating DHT, with a mean value of 12.8 ± 0.6 pg/mL. To our knowledge there are no studies in the literature regarding the circulating levels of DHT during mouse pregnancy; however, there are several reports that androgen levels increase during pregnancy in women [reviewed in Makieva et al (7)]. In women androgens seem to be “Goldilocks” factors with both deficiency and excess contributing to pregnancy- and fertility-related disorders such as polycystic ovarian syndrome (8, 9), endometriosis (10), and recurrent pregnancy loss (11).

The effects of androgens are mediated via the androgen receptor (AR), a ligand-activated transcription factor that binds T and its metabolite, 5α-dihydrotestosterone (DHT), with high affinity and specificity. In women, expression of AR in the endometrium is regulated both temporally and spatially during the normal menstrual cycle and detailed analysis of full-thickness uterine tissue sections identified endometrial stromal fibroblasts as key targets for androgen action (12). Administration of antiprogestins in women and nonhuman primates induces up-regulation of AR in the uterine epithelium, which may mediate the antiproliferative effects of these compounds (13). In the uteri of mice, quantitative in situ hybridization detected uniform labeling of *Ar* mRNA in all compartments, including the epithelium (14). A recent study reported nuclear stromal AR staining in the mouse uterus but AR was not detected in the luminal or glandular epithelium (15). A study using a transgenic mouse model of an androgen response element construct fused to a luciferase reporter revealed strong AR activity in the uterus, with the antiandrogen bicalutamide inhibiting luciferase activity (16). Female global AR knockout mice are subfertile, with a neuroendocrine and ovarian phenotype accompanied by defects in uterine development (17).

Studies in female-to-male transsexuals have reported a negative effect of androgens on endometrial proliferation in response to long-term administration of T, resulting in endometrial atrophy (18, 19). Treatment of primary human endometrial stromal cells with DHT also results in a significant reduction in both proliferation and apoptosis (12). Androgen-estrogen cross-talk has been implicated in the initiation and progression of endometrial cancers, with androgens acting in an antagonistic manner to the proliferative effects of estrogens, restricting endometrial carcinogenesis [reviewed in Gibson et al (20)]. Weihua et al (21) reported that the proliferative effects of 17β-estradiol (E_2_) in the rat uterus were blocked by either the antiestrogen tamoxifen or the antiandrogen flutamide, suggesting a functional overlap between androgen and estrogen action in this rodent species. Microarray analysis of uteri recovered from ovariectomized (ovx) mice after a single injection of DHT revealed time-dependent changes in the expression of genes involved in the regulation of cell division and organ development between 12 and 24 hours post injection (22).

Sex steroids have been shown to exert different effects in cell proliferation and apoptosis resulting from either acute or chronic stimulation [reviewed in Soto et al (23) and Groothuis et al (24)]. In the 1970s, Lee and colleagues (25, 26) recorded three phases of epithelial cell proliferation within the mouse uterus in response to continuous oral treatment with estrogen, characterized by pronounced uterine epithelial cell proliferation on days 2 and 3, basal proliferation on days 4 and 5, and a second wave of proliferation on days 19 and 20. Similar effects have been reported in prostate cancer cells in response to androgen treatment, characterized by an early proliferative response followed by a refractory period to androgen-induced proliferation (27). Taking into account this biphasic effect of androgens in controlling cell proliferation of target tissues, a short-term (24 h) and a long-term (7 d) treatment paradigm was selected to elucidate the effect of DHT on endometrial growth in a mouse model. Experiments were performed in ovx mice to avoid the confounding effect of endogenous ovarian hormones.

In the current study, we show that treatment with DHT has compartment-specific effects on the immunoexpression of AR, cellular proliferation, and expression of cell-cycle regulators and epithelial growth factors resulting in an increase in the number of uterine glands. These striking findings identify a novel role for androgens in the regulation of endometrial homeostasis.

## Materials and Methods

### Animals and treatments

Female C57BL/6J mice were purchased from Charles Rivers Laboratories and allowed to acclimate for a week with ad libitum access to food and water. Mice were maintained in accordance with United Kingdom legal requirements under licensed approval from the United Kingdom Home Office. Eight- to ten-week old female mice were ovx by dorsal bilateral ovariectomy and allowed to recover for 7 days prior to treatment to deplete endogenous sex-steroid hormones. Surgery was performed under isoflurane anesthesia followed by a postoperative analgesic, buprenorphine (0.1 mg/kg), for pain management. Mice were randomly assigned into one of four treatment groups (n = 8 per group; 32 in total). To explore the short-term and long-term effect of androgens mice received either one (24-h group) or seven (7-d group) daily sc injections of DHT (0.2 mg/mouse) or vehicle control (VC; 5% ethanol, 0.4% methylcellulose). Two hours prior to being euthanized, mice received an intra-peritoneal injection of 0.1 mL bromodeoxyuridine (BrdU) (25 mg/mL in PBS).

To measure the serum concentration of DHT after treatments, blood was collected via cardiac puncture and exsanguination from mice treated with VC (5% ethanol, 0.4% methylcellulose) or DHT (0.2 mg per mouse) for 24 hours (n = 4 per group). Serum DHT concentrations were assayed using a commercially available mouse DHT ELISA kit (Amsbio, BlueGene; Cat. No. E03D0001; assay sensitivity: 1 pg/mL). Samples were assayed in duplicate and a standard curve was generated using a four-parameter logistic curve fit; sample DHT concentrations were interpolated from the standard curve. For analysis of tissues, one uterine horn was fixed in 4% neutral buffered formalin for paraffin embedding, sectioning, and histology while the other horn was kept in RNA Save at −80°C.

### RNA extraction and reverse transcription

Total RNA was extracted from homogenized mouse uterine tissue (20 mg per sample) using TriReagent and chloroform followed by RNeasy Mini Kit (QIAGEN) column isolation and elution as per the manufacturer's instructions. RNA concentration and quality was measured using a Nanodrop ND-1000 spectrophotometer (Nanodrop Technologies) and the samples were standardized to 100 ng/μL in RNAse-free water. Reverse transcription was performed using SuperScript VILO cDNA Synthesis Kit and Master Mix (Invitrogen) per the manufacturer's instructions using a thermal cycler programmed at: 25°C for 10 minutes, 42°C for 60 minutes, and 52°C for 5 minutes. Two negative controls (no enzyme control and no RNA control) and one positive control (mouse RNA–positive control) were included for each set of RNA samples.

### Quantitative real-time PCR analysis

Quantitative real-time PCR was performed using SuperMix with Premixed ROX dye (Invitrogen), primer sets designed using the Roche Universal Probe Library Assay Design Centre (sequences in Supplemental Table 1) purchased from Eurofins (MWG Operon) and probes from the Roche Universal Probe Library Mouse Set (Roche Applied Science). Samples were assayed in duplicate and run on an ABI 7900HT Fast Real Time PCR machine using the following cycling conditions: 95°C for 10 minutes then 40 cycles of 95°C for 15 seconds and 60°C for 1 minute. Primer amplification efficiency was validated and analysis was performed using the relative standard curve method. Data were normalized to 18S and fold-change is expressed as the ratio of expression of each gene of interest in the treated groups vs the average of the VC groups (one for the 24-h and one for the 7-d time point, respectively). Statistical analysis was performed using GraphPad Prism 6.0. Data are presented as mean ± SEM. Student *t* test was used for comparison between VC and DHT groups. Criterion for significance was *P* < .05. Primer pair and probe information is provided in Supplemental Table 1.

### Immunohistochemistry and immunofluorescence

Samples were fixed in 4% neutral buffered formalin overnight at room temperature, transferred in 70% ethanol, and the tissues were processed for paraffin wax embedding. Transverse sections of 5-μm thickness were cut using a microtome (Leica RM2135) and the slides were incubated overnight at 55°C. For histology, the tissues were dewaxed, rehydrated, and antigen retrieval was performed using citrate (0.01M citrate, pH 6.0) in a decloaking chamber followed by several blocking steps with serum from the species the secondary antibody was raised in, to avoid nonspecific binding, and hydrogen peroxide was used to block endogenous peroxidase activity. Sections were incubated with the primary antibody diluted in serum blocking solution overnight at 4°C. Negative controls were included, in which the primary antibody was omitted to identify any nonspecific binding by the secondary antibody. The following day, detection was performed by using either a conjugated secondary antibody specific for the primary antibody or a polymer-based detection system (ImmPress, Vector Labs). For chromogenic immunohistochemistry, 3,3′-diaminobenzidine (DAB) (Vector) was used as a chromogen, whereas for immunofluorescence the Tyramide Signal Amplification system (PerkinElmer) was used per the manufacturer's instructions. Between incubations, washes were performed with TBS-Tween. Hematoxylin was used as a counterstain for immunohistochemistry and DAPI as a counterstain for immunofluorescence. Antibody information is provided in Table 1.

**Table 1.** Antibody Table

Peptide/Protein Target	Name of Antibody	Manufacturer, Catalog Number, and/or Name of the Individual Providing the Antibody	Species Raised in; Monoclonal or Polyclonal	Dilution Used
Androgen receptor	AR	Spring Bioscience, M4070	Rabbit monoclonal	1:300
Bromodeoxyuridine	BrdU	Fitzgerald, 20-BS17	Sheep polyclonal	1:1000
Marker of proliferation Ki67	MKi67	Abcam, Ab15580	Rabbit polyclonal	1:2000
Forkhead box protein 2	FOXA2	Santa Cruz, SC9187	Goat polyclonal	1:3000

### Image acquisition and stereology

For chromogenic immunohistochemistry, images were acquired with a Provis AX70 microscope (Olympus Optical) fitted with a Canon DS6031 camera (Canon Europe) and fluorescent images were acquired with a LSM 710 confocal microscope (Carl Zeiss) and processed with the ZEN software (Carl Zeiss). Tissue surface area was measured using a Zeiss AXIO Imager A1 (Carl Zeiss) and quantification of BrdU-positive epithelial cells and of the number of glands was performed by blinded manual counting by two independent investigators. For the calculation of cell density, sections were counterstained with DAPI and acquired images were analyzed using the image analysis software Fiji (28). In all cases, a minimum of two nonserial sections were used (n = 4–8 per treatment group). Statistical analysis was performed using GraphPad Prism 6.0. Student's *t* test was used for comparison between VC and DHT groups, with statistical significance for *P* < .05.

Unless otherwise stated all reagents for this study were purchased from Sigma-Aldrich.

## Results

### Trophic effects of androgens in the mouse uterus

To elucidate the actions of DHT in mouse uterine physiology, ovx mice were treated with either vehicle or DHT under a short- or long-term treatment regime (24 h and 7 d, respectively). ELISA analysis of serum from mice injected with VC or DHT 24 h previously had a mean DHT concentration of 180 pg/mL in the DHT group, whereas DHT concentrations in the control group were below the level of detection (< 1 pg/mL) (Supplemental Figure 1). These results seem consistent with those of Zhang et al (29), who previously demonstrated that ovx C57BL/6 female mice injected subcutaneously with 0.1 mg/mouse DHT, exhibit a peak of circulating DHT 1 hour postinjection (∼14 ng/mL), which rapidly declines during the following 24 hours.

**Figure 1.**
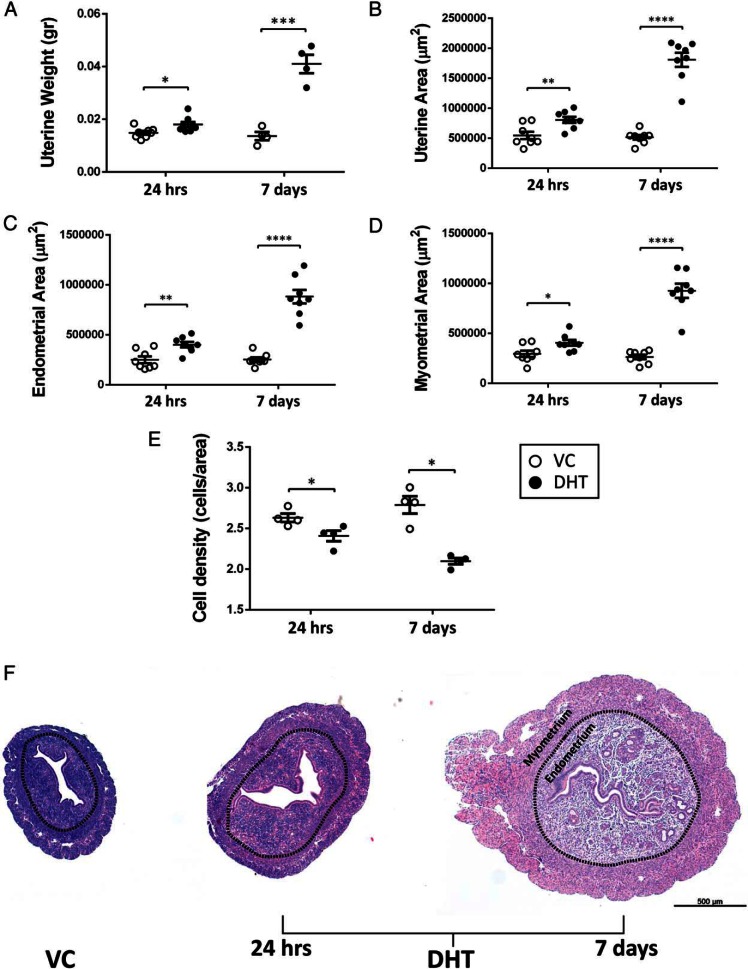
Treatment with DHT induces a trophic effect in the mouse uterus. Mice were ovx and treated with VC or DHT as described in Materials and Methods. A, At 24 h there was a modest but significant increase in uterine wet weight with DHT treatment whereas after 7 d of treatment the same effect was more pronounced. B–D, Similar effects for a time-dependent increase with DHT treatment were observed in total uterine surface area (B), endometrial area (C), and myometrial area (D). E, Treatment with DHT resulted in a significant time-dependent decrease in uterine cell density at 24 h and 7 d. F, Representative images of hematoxylin- and eosin-stained mouse uterine cross-sections after steroid treatments. All error bars represent SEM, and Student's unpaired *t* test was used for statistical analysis comparing VC- and DHT-treated samples of the respective time point (n = 4–8 per treatment group). *, *P* < .05; **, *P* < .01; ***, *P* < .001; ****, *P* < .0001. Scale bar: 500 μm.

The short-term treatment with DHT (24 h) induced a significant increase in uterine weight compared with vehicle, which was further increased when treatment was extended to 7 days (Figure 1A; *P* < .001 at 7 d). The effect of DHT on uterine weight was accompanied by significant increase of total uterine surface area (Figure 1B), endometrial area (Figure 1C), and myometrial area (Figure 1D) at both time points. Analysis of cell density suggested the change in uterine size was due to an increase in cell numbers accompanied by a significant reduction in cell density at both time points (Figure 1, E and F).

### Treatment with DHT induces epithelial AR expression in the mouse uterus

**Figure 2.**
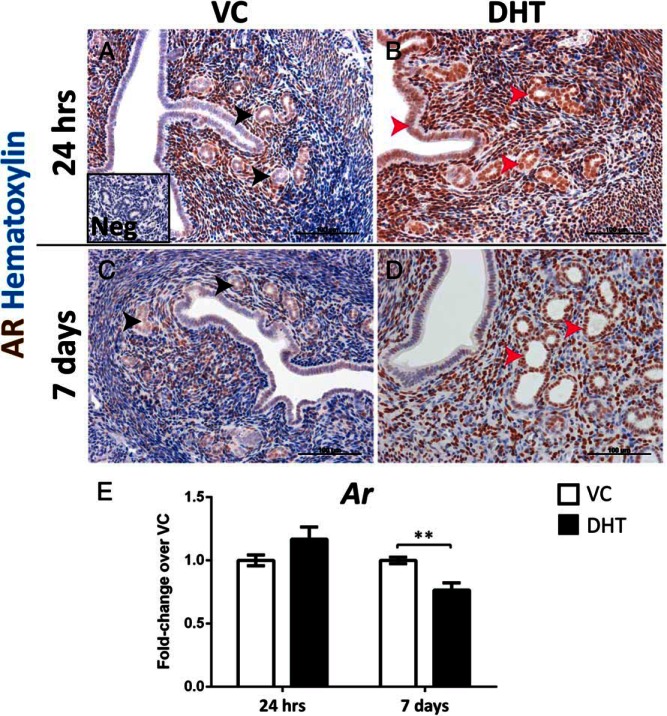
Treatment with DHT results in a marked increase of AR immunoreactivity in uterine epithelial cells accompanied by a down-regulation of *Ar* mRNA. A and C, Expression of AR was restricted to the stromal compartment and the myometrial smooth muscle cells of the vehicle groups at both 24 h (A) and 7 d (C) with glandular (black arrowheads) and luminal epithelial AR levels being low to undetectable. B and D, Treatment with DHT resulted in intense AR immunostaining in the stroma accompanied by expression of AR in the luminal and glandular epithelium after 24 h (B, red arrowheads), with the glandular epithelium retaining AR expression after 7 d of DHT treatment (D). E, Treatment with DHT for 7 d resulted in a significant down-regulation of *Ar* mRNA in the mouse uterus. All error bars represent SEM (n = 8 per treatment group). **, *P* < .01. Scale bars: 100 μm. Neg: negative control.

Positive immunoexpression of AR was detected in the stromal compartment at both 24 hours and 7 days in both vehicle and DHT-treated mice. DHT treatment induced expression of AR in the luminal and glandular epithelium at 24 hours (Figure 2B), which was absent in vehicle groups at both time points (Figure 2, A and C). In mice treated with DHT for 7 days, AR was detected in the stroma and the glandular epithelium but not the luminal epithelium (Figure 2D). Real-time PCR showed a significant decrease of *Ar* mRNA after 7 days of DHT treatment compared with vehicle (Figure 2E). Investigation of the expression pattern of estrogen receptor alpha (ERα) revealed no difference between vehicle and DHT-treated groups at both time points (Supplemental Figure 2).

### Treatment with DHT induces epithelial proliferation in the mouse uterus

**Figure 3.**
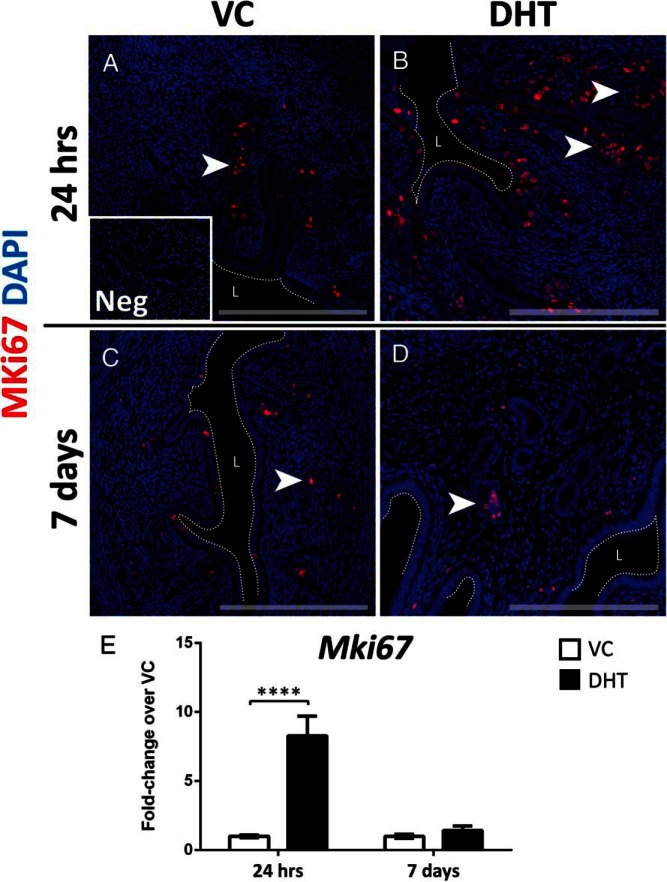
Short-term treatment with DHT increases MKi67 expression in uterine epithelial cells. A and C, VC-treated groups displayed nuclear immunostaining for MKi67 (in red staining) in a small proportion of epithelial cells (arrowheads indicate MKi67-positive epithelial cells) at both 24 h (A) and 7 d (C). B, Treatment with DHT for 24 h induced marked expression of MKi67 in the luminal and glandular epithelium, whereas after 7 d MKi67 staining did not differ from VC-treated groups (D). E, Real-time PCR revealed a significant increase in *Mki67* mRNA levels after DHT treatment at 24 h, with no difference observed at 7 d between vehicle and DHT. All error bars represent SEM (n = 8 per treatment group). ****, *P* < .0001. Scale bars: 200 μm. L, lumen; Neg, negative control.

Immunofluorescence was performed with an antibody targeting the proliferation marker MKi67, which detects all cells in interphase or undergoing mitosis. Staining identified extensive proliferation of luminal and glandular epithelial cells after treatment with DHT at 24 hours compared with vehicle (Figure 3, A and B; white arrowheads). Only a small number of epithelial cells were positive for MKi67 in both vehicle- and DHT-treated groups after 7 days of treatment (Figure 3, C and D; white arrowheads). The immunostaining for MKi67 in the stroma was minimal for all treatment groups. DHT induced a significant increase of *Mki67* mRNA levels after 24 hours of treatment compared with vehicle, whereas at 7 days there was no significant change between the two treatment groups (Figure 3E), consistent with MKi67 staining detected by immunofluorescence.

**Figure 4.**
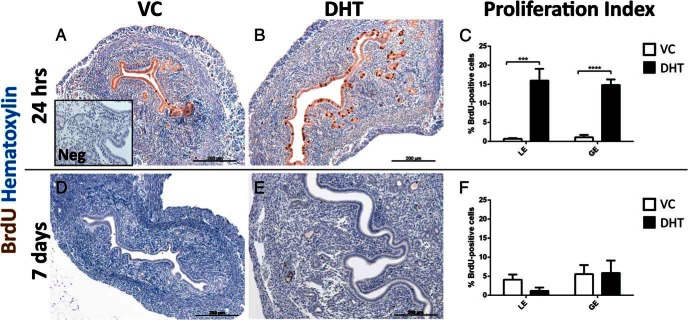
DHT stimulates transient epithelial cell proliferation in both glandular and luminal compartments. A and B, With VC treatment, BrdU was detected in very few epithelial cells after 24 h, whereas treatment with DHT induced pronounced luminal and glandular epithelial nuclear BrdU incorporation. C, Quantification of BrdU-positive cells revealed a significant increase of luminal (LE) and glandular (GE) epithelial proliferation with DHT after 24 h of treatment. D–F, After 7 d of treatment, proliferation index showed no significant difference between VC and DHT groups. All error bars represent SEM (n = 8 per treatment group), ***, *P* < .001; ****, *P* < .0001. Scale bars: 200 μm. Neg: negative control.

DNA incorporation of BrdU was used as a measure of the proliferative state of cells during the 2 hours prior to animal euthanasia. Staining with an anti-BrdU antibody revealed a limited number of positive epithelial cells in the vehicle-treated groups for both time points (Figure 4, A and D). Treatment with DHT induced extensive proliferation of both the glandular and luminal epithelium at 24 hours (Figure 4B). Very low proliferation was detected in the stromal compartment. This was also confirmed by assessment of the proliferation index of epithelial cells; there was a significant increase in the proliferation of luminal and glandular epithelial cells between the vehicle and DHT treatment groups at 24 hours (Figure 4C). That was absent after 7 days (Figure 4, D–F).

### DHT regulates genes necessary for proliferation, cell-cycle regulation, and stromal-epithelial cross-talk in the mouse uterus

Real-time PCR analysis identified dynamic changes in the expression of several genes associated with cell-cycle regulation and stromal-epithelial cross-talk following treatment with DHT.

**Figure 5.**
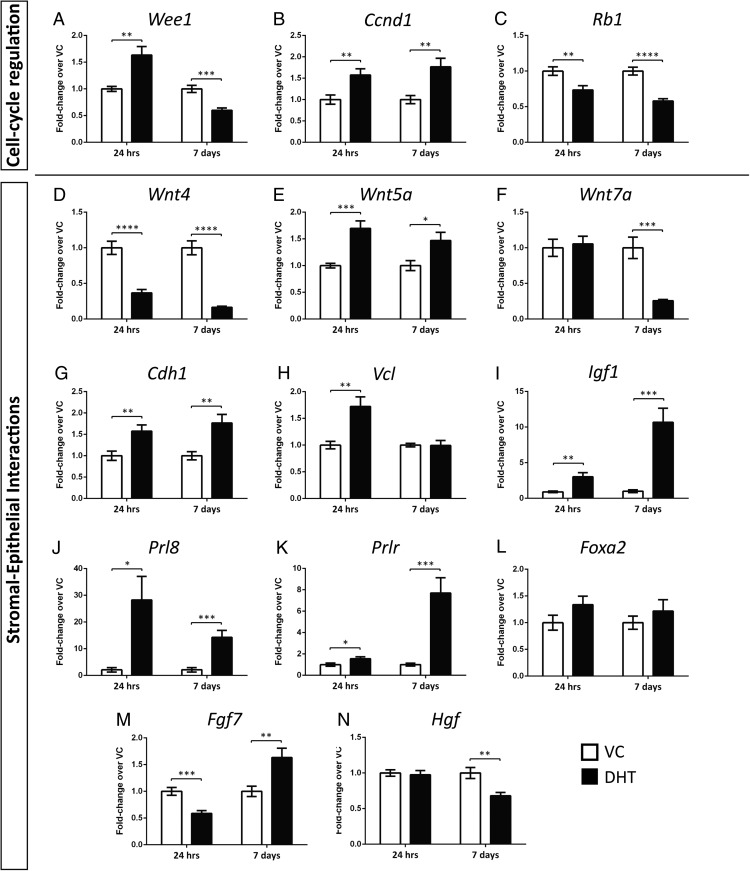
Treatment with DHT has time-dependent effect on concentrations of mRNAs encoded by genes implicated in regulation of the cell cycle and stromal-epithelial cross-talk. Real-time PCR analysis of whole-tissue uterine homogenates identified altered expression of cell-cycle regulators and proliferation markers (*Wee1* [A], *Ccnd1* [B], *Rb1* [C]) and genes involved in stromal-epithelial interactions and epithelial growth factors (*Wnt4* [D], *Wnt5*α [E], *Wnt7*α [F], *Cdh1* [G], *Vcl* [H], *Igf1* [I], *Prl8* [J], *Prlr* [K], *Foxa2* [L], *Fgf8* [M], *Hgf* [N]). For all RT-PCR analyses, n = 8 per treatment group per time point were used and all data were normalized to the housekeeping gene 18s. All error bars represent SEM, *, *P* < .05; **, *P* < .01; ***, *P* < .001; ****, *P* < .0001.

*Wee1* expression was significantly up-regulated by DHT after 24 hours, followed by a significant down-regulation at 7 days (Figure 5A). *Ccnd1* expression was significantly increased by DHT at 24 hours followed by a significant decrease after 7 days (Figure 5B). *Rb1* expression was significantly down-regulated by DHT at both time points (Figure 5C).

Wnt signaling, involved in stromal-epithelial cross-talk in the uterus, was significantly affected by treatment with DHT. *Wnt4* expression was significantly down-regulated after treatment with DHT for 24 hours and 7 days (Figure 5D). *Wnt5a* mRNA concentration was significantly increased by DHT at both time points (Figure 5E), whereas *Wnt7a* expression was down-regulated by DHT only at 7 days (Figure 5F).

*Cdh1*, which is expressed in the uterine epithelium was significantly up-regulated by DHT at both 24 hours and 7 days (Figure 5G), whereas expression of the adherens junction component vinculin (*Vcl*) involved in intercellular adhesion and signaling displayed a significant up-regulation by DHT at 24 hours, with no significant change observed between vehicle and DHT at 7 days (Figure 5H). Expression of the putatively androgen-regulated epithelial growth factor *Igf1* was significantly up-regulated by DHT at both time points compared with vehicle treatments, reaching an average 10-fold increase at 7 days (Figure 5I). *Prl8,* whose role has been reported in gland formation in the mouse uterus, was significantly up-regulated more than 20-fold at both time points (Figure 5J), with a similar pattern observed for its receptor (*Prlr*; Figure 5J). The transcription factor *Foxa2*, which is exclusively expressed in uterine glands exhibited a trend for an increase after DHT treatment at 24 hours (*P* = .14) but the changes at both time points did not reach statistical significance (Figure 5K). Furthermore, expression of the epithelial growth factors *Fgf7* (Figure 5L) and *Hgf* (Figure 5M) was significantly altered after treatment with DHT.

### A role for androgens in regulating gland formation in the mouse uterus

**Figure 6.**
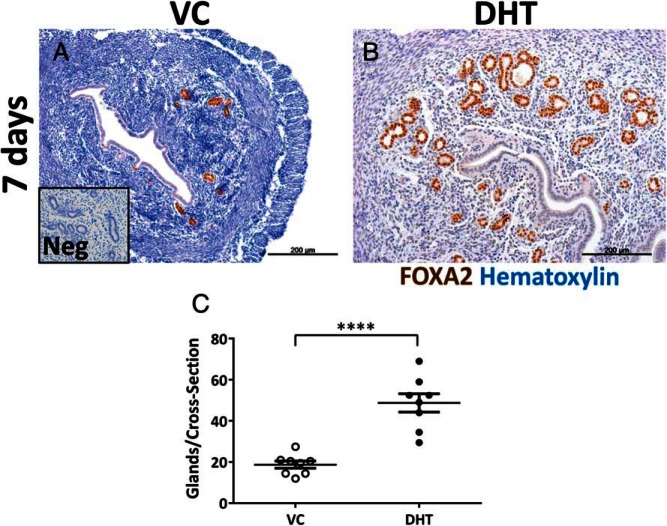
Androgen treatment stimulates the glandular compartment of the mouse uterus. Immunohistochemistry for the gland-specific transcription factor FOXA2 revealed more glands after DHT treatment (B) compared with VC (A) at 7 d. Quantification of the glands (C) demonstrated a significant increase in the total number of glands per cross-section after DHT treatment compared with vehicle. All error bars represent SEM (n = 8 per treatment group). ****, *P* < .0001. Scale bars: 200 μm. Neg: negative control.

Histological examination of uterine sections at 7 days suggested more glands with DHT treatment. FOXA2 is a transcription factor expressed exclusively in the glands in the mouse uterus and FOXA2 immunohistochemistry was performed to quantify the number of glands between the treatment groups (Figure 6, A and B). A significant increase in the total number of glands was observed with DHT compared with vehicle treatment verifying the qualitative observations (Figure 6C). To compare the effect of DHT with that with E_2_ in glandular expansion, ovx mice treated with an E_2_ pellet for a week were used as a comparator. Staining for FOXA2 and quantification of glands demonstrated fewer glands in the E_2_ group compared with the DHT-treated group accompanied by a weaker staining pattern (Supplemental Figure 3, A and D). Real-time PCR identified a significant reduction in *Foxa2* expression levels between E_2_ and DHT-treated groups (Supplemental Figure 3).

## Discussion

In this study, we have demonstrated that androgens induce epithelial proliferation resulting in increased numbers of endometrial glands in the adult mouse uterus. Uterine glands are essential for uterine receptivity and implantation and secrete factors which are necessary for the survival and development of the conceptus. Our results suggest that homeostatic balance of androgens and the local steroidogenic environment could be important physiological drivers of gland formation during endometrial remodelling in early pregnancy. Animal models in which uterine gland development is disrupted exhibit compromised fertility and altered expression of decidualization factors (30, 31), highlighting the potential for prospective therapeutic targeting of androgen signaling in the uterus for fertility-associated disorders (32). Although a trophic effect of androgens in the rodent uterus has previously been reported, we identify for the first time the molecular mechanisms involved and selective effects of androgens on distinct tissue compartments within the uterus. Notably, we describe for the first time that DHT promotes gland formation in the adult mouse uterus through directly stimulating glandular epithelial proliferation and by driving expression of regulators of cell cycle and stromal-epithelial interactions. The novel data generated in the current study provide new insights into the role of androgens in the regulation of endometrial function.

In the uterus, sex steroid action occurs via direct steroid hormone receptor signaling in target cells and indirect paracrine effects on neighboring cellular compartments. Such examples of intercellular paracrine signaling include the stromal-derived secretion of IGF-1 and WNT5a binding on epithelial receptors controlling proliferation and gland formation, respectively. Thus, regulation of the complex stromal-epithelial interactions resulting from androgen action can be investigated using in vivo model systems, rendering rodents invaluable models. To investigate the effect of androgen treatment in the steroid-depleted mouse uterus, we treated ovx mice with DHT at a supraphysiological dose (0.2 mg/mouse) previously shown to elicit uterine responses (29, 33). Although the DHT concentrations generated in our experimental conditions are above the physiological levels detected in cycling female mice (6), this dose is consistent with those used in several previous studies investigating the direct action of DHT in the rodent uterus (22, 34) and adipose tissue (29, 35).

In this study, DHT induced a time-dependent increase in uterine weight in agreement with previous reports in rats treated with DHT or the synthetic androgen mibolerone, where a dose- and time-dependent increase in uterine weight after androgen treatment was detected in that species (34). In the present study, increases in total uterine area, endometrial area, and myometrial area accompanied by a significant decrease in cell density, possibly due to water imbibition, were detected, demonstrating the trophic effects of DHT in both uterine compartments in the mouse. Although trophic effects are also induced by estrogens in the rodent uterus, in the current study uterine morphology after DHT treatment lacked the characteristic distended phenotype, edema, and thinness of the endometrial layer induced by E_2_ (36, 37) consistent with a unique effect of androgens in regulating uterine physiology that is distinct from that of estrogens. Early studies using in vitro cell-free systems and in vivo rodent models have demonstrated interaction between T and DHT with ER, leading to nuclear translocation of ER, whereas cotreatment with antiestrogens but not antiandrogens blocked this effect (38, 39). However, a later study demonstrated that the in vivo doses of DHT required to elicit ER cytoplasmic depletion and nuclear translocation in the rat uterus are very high (5–10 mg), suggesting that the uterine trophic effects of DHT observed in our study are unlikely to be mediated by ER (40). In addition, ovx female rats cotreated with DHT and the AR antagonist bicalutamide exhibited a complete suppression of DHT-induced expression of *Igf1*, a growth factor whose up-regulation is both ERα- and AR-controlled (34). This finding, together with the fact that putative ERα target genes are not induced after treatment with DHT in the mouse uterus (34), suggest more of a functional interaction between AR and ERα than a physical one. Notably, in a study describing the development of a uterine glandular epithelial specific AR-knockout mouse model (ugeARKO), uterine parameters (uterine weight and surface area) of adult ugeARKO female mice were within the normal range but androgen-induced uterine growth in ovx mice was impaired (41), consistent with the results in the present study, which describe androgenic induction of uterine growth via epithelial AR.

Given that androgens promote growth of the rodent uterus, we investigated expression of proliferation markers in the tissue to elucidate compartment-specific effects. Assessment by immunofluorecence of the proliferation marker MKi67, which detects all actively proliferating cells (cells not in G_0_ of the cell-cycle), revealed extensive proliferation within the uterine epithelium that was associated with a significant increase in concentrations of *Mki67* mRNA after 24 hours that was also accompanied by nuclear translocation of cyclin D1 in the epithelium (data not shown). The proliferative effect of DHT was confirmed by assessing incorporation of BrdU, which revealed extensive proliferation in the epithelial compartment but not in the stroma after treatment with DHT. Notably, increased proliferation was only detected after 24 hours of treatment consistent with DHT inducing rapid changes in the tissue. Our findings are further complemented by Terada et al (42), who reported that treatment of ovx E_2_-primed mice with DHT significantly decreased the apoptotic index of the uterine epithelium, thus arguing for an inhibitory effect of DHT on cell death on the mouse uterine epithelium. Furthermore, the effects of DHT partially mimic those seen after treatment with E_2_ where maximal epithelial proliferation rate is observed after 21 hours of E_2_ treatment followed by a decline in proliferation and increased apoptosis after repeated E_2_ injections (43).

Treatment of ovx mice with DHT resulted in a striking increase in AR immunoreactivity in the uterine stroma accompanied by induced expression of AR in glandular and luminal epithelial cells. Previous studies have demonstrated that while androgens decrease AR transcription, they positively modulate translation efficiency and stability of the AR protein, thus increasing AR protein levels (44–47). These findings are in agreement with our data in which we detected a significant decrease of *Ar* mRNA following treatment with DHT for 7 days accompanied by an increase of AR protein levels. As already mentioned, previous studies have reported that androgenic effects in the mouse uterus can be mediated via ERα signaling (21, 34), with extensive cross-talk between the two receptors (40, 48). However, no change was observed in the expression pattern of ERα after DHT treatment, suggesting that if any functional interaction occurs between AR and ERα following DHT treatment it is not due to altered ERα levels.

Taking together the uterine epithelial proliferation following DHT treatment with the known role of androgens in controlling cell-cycle progression in the prostate (49) and the mouse uterus (22), transcript expression of cell-cycle regulators was analyzed by real-time PCR. The multifunctional cell-cycle regulator Wee1 controls chromatin integrity and entry into mitosis by regulating the transcription of histones and forming active complexes with factors that control cell-cycle progression [reviewed in Mahajan et al (50)]. DHT induced up-regulation of *Wee1* at 24 hours, consistent with the actively proliferating epithelial compartment at that time point, whereas its expression after 7 days of DHT treatment was significantly reduced compared with VC, suggesting a negative feedback mechanism restoring tissue proliferation to basal levels. The same expression pattern was observed for *Ccnd1* (cyclin D1), whose levels and nuclear localization have been shown to be regulated by E_2_ in mouse uterine epithelial cells (51). Expression of the cell-cycle inhibitor and tumor suppressor *Rb1* was significantly down-regulated by DHT at both time points, consistent with increased proliferation and growth of the uterus detected in response to DHT. Given that the changes seen in uterine size reflect changes in all cellular compartments (including the stroma) additional studies are required to investigate whether changes in stromal proliferation occur in response to DHT across other time points post androgen administration. The results of the current study extend previous transcriptional analyses of samples generated from DHT-treated isolated human endometrial stromal cells which demonstrate AR-dependent repression of cell cycle regulators (52) and DHT-dependent decreases in both proliferation and apoptosis (12). Taken together these results suggest androgens may impact on the stromal compartment to inhibit stromal cell apoptosis, rather than promoting proliferation, in addition to the direct effects on the uterine epithelial proliferation described in the current study.

accompanied by down-regulationaccompanied by down-regulation involved in stromal-epithelial interactions and organ patterning during neonatal and postnatal uterine development. *Wnt4* and *Wnt5a* are expressed by stromal fibroblasts and *Wnt7a* by epithelial cells with paracrine and autocrine signaling controlling cell fate and differentiation of the uterus (53). We observed significant changes in the expression pattern of these factors, with *Wnt4* and *Wnt7a* being significantly down-regulated and *Wnt5a* significantly up-regulated after DHT treatment. Studies with mice have demonstrated that all three Wnts are required for gland formation in the uterus and knockout mice for these Wnts have compromised fertility (54–56). In addition, administration of either progesterone or the synthetic estrogen diethylstilbestrol during an early postnatal time window inhibits endometrial formation of glands (adenogenesis) in the mouse uterus (3, 57). Similarly, cadherin 1 (*Cdh1*) has been to shown to be vital for endometrial differentiation of the uterine epithelium, whereas loss of *Cdh1* in the uterus leads to absence of glands, a disorganized luminal epithelium and up-regulation of *Wnt4a* with concomitant down-regulation of *Wnt5a* in the neonatal mouse uterus (58). In our study, treatment with DHT induced a significant up-regulation of *Cdh1* at both 24 hours and 7 days, accompanied by down-regulation of *Wnt4a* and up-regulation of *Wnt5a*, suggesting that androgens in the mouse uterus positively regulate formation of glands.

Trophic effects in tissues are often accompanied by tissue remodelling and that led us to investigate the expression of vinculin (*Vcl*), an adherens junction component involved in cell-cell adhesion, whose expression is androgen regulated in the prostate (59). *Vcl* was significantly up-regulated after 24 hours of DHT treatment, and by 7 days its expression was the same as the vehicle, indicating an initial phase of alteration in cell adhesion properties followed by restoration to basal levels. In addition, this is the first evidence of androgenic regulation of *Vcl* in the uterus.

To corroborate the increased epithelial proliferation after treatment with DHT, expression of epithelial growth factors was assessed. IGF-1 is predominantly produced by mouse endometrial stromal cells following E_2_ treatment and signals to the epithelium to proliferate (51) and previous studies have reported that the *Igf1* promoter contains androgen response elements (60). In the current study, a significant increase in the expression of the epithelial growth factor *Igf1* was detected after 7 days of DHT treatment. Notably, Nantermet et al (34) reported that DHT did not induce uterine hypertrophy in mice lacking a functional estrogen receptor (ERα-knockout mice), suggesting that ERα is required to mediate DHT responses. However, Klotz et al (61) have previously reported that ERα expression is necessary to mediate the proliferative effect of IGF-1 in the mouse uterus. Thus, taken together with the data in the current study, these results are consistent with DHT also mediating indirect effects on proliferation in the uterus through stromal-epithelial cross-talk following induction of the IGF-signaling pathway. The stromal-derived epithelial growth factors FGF7 and HGF both mediate stromal-epithelial interactions in the uterus by promoting epithelial proliferation with important roles in epithelial morphogenesis (62). Although progesterone administration in the neonatal mouse uterus inhibits uterine gland formation accompanied by a significant reduction in the expression of *Fgf7* and a significant increase in the expression of *Hgf* (62), in our study, treatment with DHT induced the opposite effect by significantly increasing *Fgf7* mRNA levels and decreasing *Hgf* mRNA levels.

The most striking result observed was the effect of DHT on uterine glands. In control uteri, AR expression was not detected in glandular epithelial cells; however, DHT treatment induced AR expression and increased glandular epithelial proliferation. Quantification of FOXA2-positive glands demonstrated more uterine glands in the DHT-treated mouse uterus, an effect that was unique to DHT, as treatment with E_2_ did not produce this effect. FOXA2 is expressed exclusively in the uterine glandular epithelium of mice and *Foxa2*-uterine-knockout mice fail to develop glands (30). In the current study, DHT induced changes in expression of a number of factors that promote adenogenesis in adult mice. Although expression of *Foxa2* mRNA in whole tissue homogenates was not significantly different between control and DHT-treated mice, treatment with E_2_ significantly decreased *Foxa2* mRNA concentrations highlighting the importance of androgen-estrogen balance in the regulation of this cellular compartment in the uterus. Taken together, these data are consistent with DHT regulating gland formation in the adult mouse uterus by inducing a transcriptional program that results in de novo adenogenesis.

In the present study, treatment of mice with DHT revealed androgen-specific effects on endometrial architecture characterized by a rapid increase in proliferation of epithelial cells lining the glands and significant DHT-dependent changes in expression of genes associated with cell-cycle regulation and stromal-epithelial cross-talk. These results suggest that any endocrine pathology that resulted in significant deviation from normal intra-tissue concentrations of androgens could have a long-term effect on the abundance of glandular epithelial cells, with consequent effects on glandular secretions and epithelial-stromal cell cross-talk required to support establishment of pregnancy in women.
